# Diagnostic delay in late‐onset Pompe disease among Chinese patients: A retrospective study

**DOI:** 10.1002/jmd2.12404

**Published:** 2023-12-20

**Authors:** Dongyue Yue, Kexin Jiao, Xingyu Xia, Jialong Zhang, Bochen Zhu, Lingchun Liu, Kunzhao Du, Mingshi Gao, Nachuan Cheng, Ningning Wang, Sushan Luo, Jianying Xi, Jiahong Lu, Chongbo Zhao, Wenhua Zhu

**Affiliations:** ^1^ Department of Neurology Jing'an District Center Hospital of Shanghai Shanghai China; ^2^ Department of Neurology Huashan Hospital, Fudan University Shanghai China; ^3^ National Center for Neurological Disorders (NCND) Shanghai China; ^4^ Huashan Rare Disease Center Shanghai Medical College, Huashan Hospital, Fudan University Shanghai China; ^5^ The First People's Hospital of Yunnan Province Yunnan China; ^6^ Jinshan Hospital Center for Neurosurgery, Jinshan Hospital, Institute for Translational Brain Research, State Key Laboratory of Medical Neurobiology, MOE Frontiers Center for Brain Science Fudan University Shanghai China; ^7^ Department of Pathology Huashan Hospital, Fudan University Shanghai China

**Keywords:** China, diagnostic delay, late‐onset Pompe disease, patient journey

## Abstract

Surveys and retrospective studies have revealed considerable delays in diagnosing late‐onset Pompe disease (LOPD) in China, where the contributing factors remain poorly represented. Our study analyzed the diagnostic journey of 34 LOPD patients seen at our neuromuscular clinic from 2005 to 2022. We defined diagnostic delay as the time from the onset of the first relevant symptoms and laboratory findings suggestive of LOPD to the eventual diagnosis, and we constructed a correlation matrix to assess relationships among these variables. The cohort consisted of 34 patients with an equal male‐to‐female ratio, and the mean age at diagnosis was 27.68 ± 10.03 years. We found the median diagnostic delay to be 5 years, with a range of 0.3 to 20 years, with 97.1% having been misdiagnosed previously, most commonly with “Type II Respiratory insufficiency” (36.7%). Notably, patients at earlier onset (mean age, 18.19 years vs. 31 years; *p* < 0.005) tended to have higher creatine kinase (CK) levels. Furthermore, 92.6% reported difficulty in sitting up from a supine position since childhood. Our research emphasizes the role of early indicators like dyspnea and difficulty performing sit‐ups in adolescents for timely LOPD diagnosis and treatment initiation. The importance of early high‐risk screening using dried blood spot testing cannot be overstated.

**Synopsis**

This study illustrated how the initial symptoms influenced the referral flowchart that a wide variety of specialties were involved as the initial referral department. It provides the clue of how further medical education should exerted. In light of these findings, we strongly advocate for the early screening of GAA enzymatic activity through dried blood spot (DBS) testing in high‐risk populations.

## 1 INTRODUCTION

Pompe disease, also known as glycogen storage disease type II, is a rare autosomal‐recessive disorder caused by a deficiency of acidic α‐1,4‐glucosidase (GAA) which is crucial for converting glycogen to glucose within lysosomes.^1^ Incidence rates appear to differ across ethnic groups, ranging from 1 in 14 000 to 1 in 300 000.^2^, ^3^ The phenotypic spectrum broadly ranges from classic infantile Pompe disease presenting with cardiac hypertrophy, respiratory dysfunction and floppiness to attenuated, late‐onset forms that are more benign and more heterogeneous with respiratory and skeletal muscle involvement.^4^

Late‐onset Pompe disease (LOPD) manifests in juveniles or adults as a slowly advancing condition primarily characterized by progressive muscular weakness and respiratory difficulties. Its symptoms can mimic those of other muscular disorders, such as limb‐girdle muscular dystrophies, other glycogen storage diseases, and inflammatory myopathies.^5^, ^6^ Morbidity and mortality are mainly related to ventilatory insufficiency, while the first clinical manifestations, such as exercise intolerance, muscle pain or even isolated hyperCKemia, can be easily overlooked.^7^ Consequently, misdiagnosis or significant diagnostic delays are common.^8^ Patients with LOPD are generally diagnosed 12 (1–40) months after the onset of the first symptoms.^9^ In mainland China, the average diagnostic delay spans approximately 7.2 years.^10^

Previous studies indicate that an earlier onset of LOPD often correlates with shorter times to diagnosis.^9^ At present, Pompe disease is treatable but not curable. Enzyme replacement therapy (ERT) is less effective in older juveniles and adults, largely due to the delayed diagnosis.^11^, ^12^ Recognition of the disease remains inadequate, leading to underdiagnosis and significant delays.^13^, ^14^ Chinese patients with LOPD typically experience symptom onset in their second decade, with a rapid progression likely linked to specific GAA gene mutations.^15^ The risk factors contributing to diagnostic delays in the Chinese LOPD populations remain under‐researched. Our study aims to identify these factors to promote earlier diagnosis and better patient outcomes.

## 2 PATIENTS AND METHODS

### 2.1 Patients

LOPD patients diagnosed at the Neuromuscular Clinic of Huashan Hospital, Fudan University, a referral Neuromuscular Center covering East China, were included in the study from July 2005 to September 2022. To meet the diagnostic criteria according to the Expert Consensus on diagnosis and treatment of Chinese LOPD,^16^ the patient should have at least two of the following: (1) decreased GAA enzymatic activity; (2) molecular analysis confirming two pathogenic *GAA* mutations; and (3) muscle pathology showing vacuolar changes with excessive glycogen. For each patient, clinical history and laboratory data were collected in a case report form (CRF). The project was approved by the Institutional Ethics Committee of Fudan University. Informed consent was obtained from all living patients and family members involved in this study.

### 2.2 Patient journey analysis

The CRF included parameters such as demographic information, presenting symptoms, medical history details, family history, biochemical tests including GAA enzymatic activity, molecular genetic tests confirming GAA mutations, and muscle pathology reports. The patients' journey and diagnosis were based on the medical records provided by the patients or their attending doctors. The ‘age of onset’ was defined as the age at which the patient began to be aware of the symptoms, while the ‘age at diagnosis’ was when they were clinically diagnosed with LOPD. ‘Diagnostic delay’ was the period between the onset and diagnosis, with delays under 12 months being converted into years for consistency. In terms of symptoms, difficulties in raising head at a supine position or difficulty in sit‐ups were categorized as axial muscle weakness (AMW). Shortness of breath after exercise or at night was categorized as dyspnea. Asymptomatic hyperCKemia was defined as consistent elevated serum creatine kinase (CK) level above two times of upper normal limit (UNL) for over 2 weeks without other evident symptoms and signs. Symptoms categorized as limb‐girdle muscle weakness including difficulty in walking a long distance, climbing stairs, or as mild as a waddling gait. Respiratory insufficiency is defined as forced vital capacity less than 80% of predicted.

### 2.3 Statistical analysis

We performed statistical analysis using SPSS statistical software (versionm27; SPSS, Chicago, IL). Categorical variables are presented as the number (percentage) of participants, and continuous variables are presented as the mean and SD; or, for those data with a skewed distribution, as the median (25th percentile, 75th percentile). The cohort was stratified into two groups according to the CK level at diagnosis: the CK_low_ group lower than two times of UNL, and the CK_high_ group no less than two times of UNL. Differences in patient characteristics between these strata were tested for statistical significance with the use of the chi‐square test or the Fisher exact test for categorical data and the Student *t* test or Mann–Whitney test for continuous variables. Frequency differences were compared using *χ* ^2^ test or Fisher's exact test, where appropriate. *p* values less than 0.05 were considered to indicate statistical significance. Data were analyzed using SPSS statistical software (version 27.0; SPSS; Chicago, IL), GraphPad Prism 9 and R studio.

## 3 RESULTS

### 3.1 Demographic characteristics

Thirty‐four patients with LOPD were enrolled in this study (Table S1). Biochemical analysis confirmed reduced α‐1,4‐glucosidase activity in all 18 patients tested. Muscle biopsies performed on 33 patients revealed that 28 (84.8%) exhibited vacuolar myopathy with abundant glycogen deposits, identified by Periodic Acid‐Schiff (PAS) staining. All the patients harbored two *GAA* mutations. The c.2238G>C (p.W746C) variant was the most prevalent, found in 32.4% (22/68) of alleles. Besides the previously reported c.568C>G (p.R190G) and c.1928G>C (p. G643A),^17^ two novel variants, c.1444C>T (p.P482S) and c.521A>G (p.E174G), were identified in two independent patients, respectively. They were categorized as “Likely pathogenic” and “uncertain significance” according to established criteria, respectively (Table S2).

### 3.2 Patient journey

The most common early symptoms reported were limb‐girdle muscle weakness (LGMW, 67.6%). Dyspnea (14.7%) are less frequent as initial symptom although upon diagnosis most of patients had respiratory insufficiency. Axial muscle weakness was also less frequently reported as initial symptom (12%). Asymptomatic hyperCKemia was only noticed in two patients (2.9%), one of which carried the common Caucasian variant IVS1 (Table S1). Limb‐girdle weakness is a non‐specific symptom which can be found in a wide range of diseases. Therefore, a large variety of specialties were involved as the first referral department. Patients with dyspnea often went to pulmonary and cardiovascular consultants instead of neurologists. Back pain and stiffness of spine accompanied with axial muscle weakness led to orthopedic consultation. One patient with axial muscle weakness with diarrhea and weight loss went to the gastroenterology department first.

**FIGURE 1:**
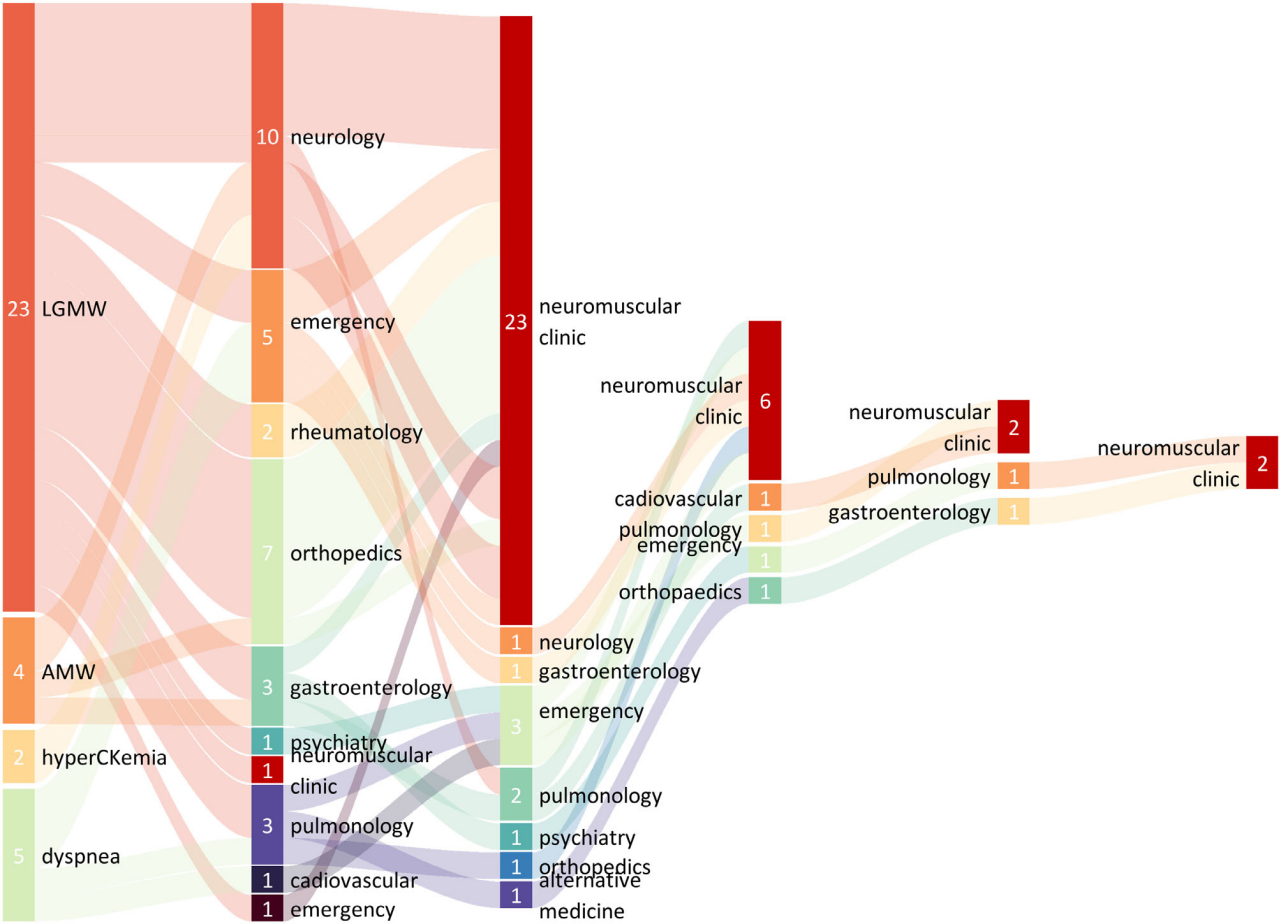
Initial symptoms and flowchart of patient referrals until diagnosis.

**FIGURE 2:**
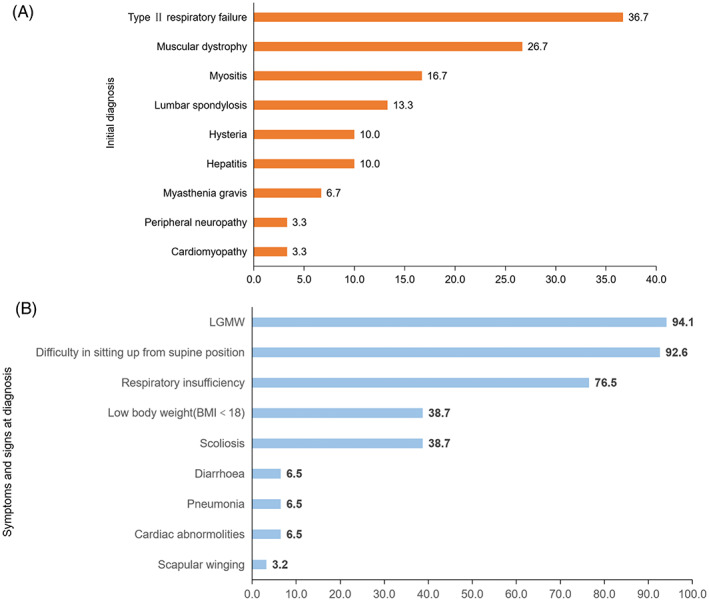
(A) Misdiagnoses prior to patient confirmation of diagnosis, (B) Symptoms and signs at diagnosis.

The median time to diagnosis was 5 years, with a range of 0.5–20 years. The majority of the patients (33/34, 97.1%) had initially been misdiagnosed, except for patient 17, who was straightly referred to our center by an experienced family doctor. Many had consulted with two to five different specialists, including those in emergency medicine (30.3%), orthopedics (33.3%), neurology (33.3%), and respiratory medicine (21.2%), prior to being referred to a neuromuscular center (Figure 1). The most common misdiagnoses were “Type II respiratory failure” (36.7%), followed by “muscular dystrophy” (26.7%),”myositis” (16.7%), and “lumbar spondylosis” (13.3%) (Figure 2A).

At diagnosis, 94.1% (32/34) exhibited LGMW, primarily in the lower limbs. Respiratory insufficiency was noted in 76.5% (26/34) of the patients. Dyspnea was even presented as the only symptom in one patient. The median CK level at diagnosis was 624 U/L [interquartile range (IQR): 314.75–1016.25 U/L], with 17.6% (6/34) of patients presenting with normal serum CK levels. None of the six patients had any previous medical history of hyperCKemia or elevated aspartate transaminase level before. Aside from seven individuals who had never attempted sit‐ups, 92.6% (25/27) could not complete sit‐up tests since childhood. Patients 10 and 27 managed sit‐ups in adolescence at a normal rate but failed to do so in adulthood. This is an easily overlooked sign as it has no impact on daily activities and exercise in early stage (Figure 2B).

### 3.3 Risk factors for diagnostic delay

**FIGURE 3:**
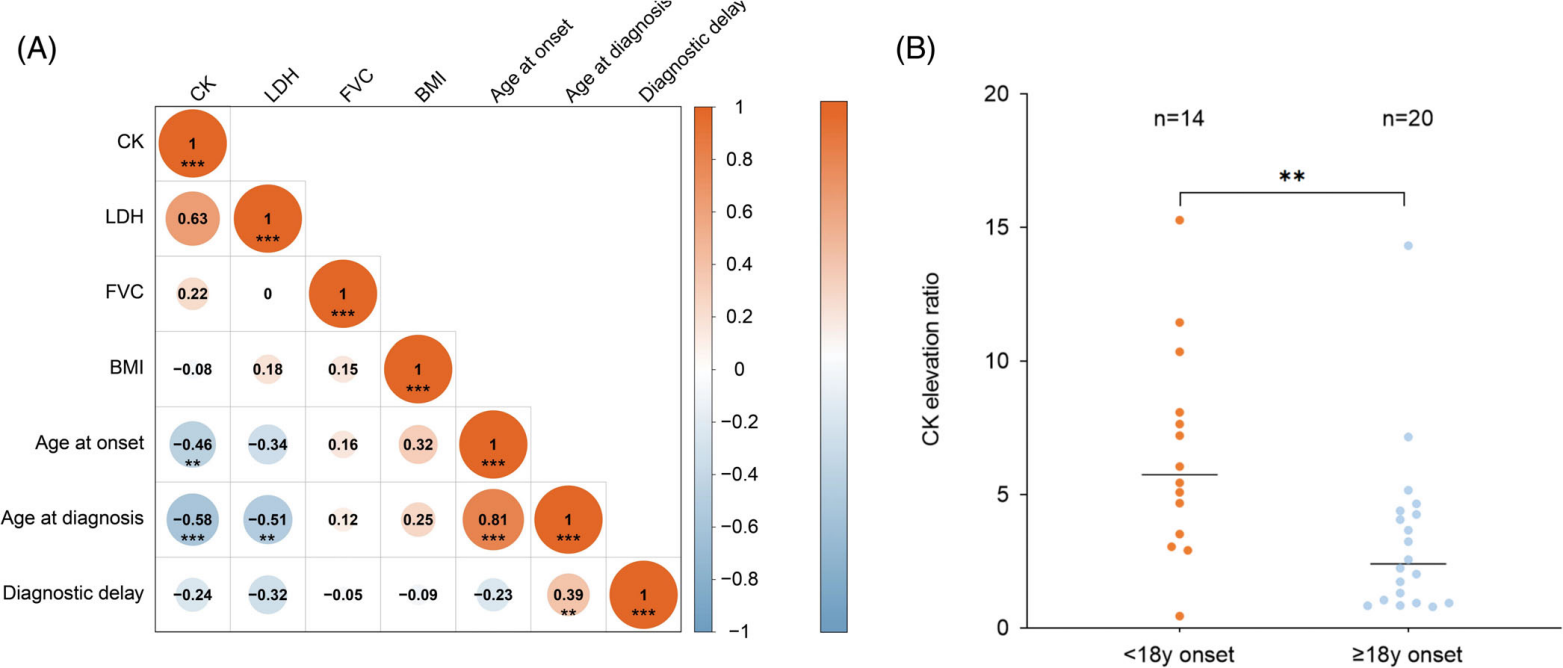
(A) Correlation matrix of all variants in this study (Pearson correlation test). (B) Comparison of CK elevation ratios in patients with LOPD based on age at onset. BMI, body mass index; CK, creatine kinase; EMG, electromyography; FVC, forced vital capacity. *P<0.05; **P<0.01; ***P<0.001.

Given the negative correlation between CK levels and diagnostic delay, patients were stratified into CK_low_ (9 patients) and CK_high_ (25 patients) groups at diagnosis. The CK_high_ group was younger at disease onset (average 18.2 years vs. 31.0 years) and diagnosis (average 23.3 years vs. 39.7 years) compared to the CK_low_ group with significant differences (*p* < 0.005 and *p* < 0.001, respectively). This group also more frequently presented with LGMW as an initial symptom (84% vs. 44%; *p* = 0.034) and less often with dyspnea (8% vs. 44%; *p* = 0.031). The CK_low_ group tended to have a longer diagnostic delay compared with the CK_high_ group (average 7 years vs. 3 years), though no statistical significance was reached (*p* = 0.433) (Table 1, Figure 3A). A further analysis showed that childhood‐onset LOPD patients (onset before 18 years) had a higher average CK level compared to adult LOPD (onset at or after 18 years) (median 6.62 UNL vs. 3.31UNL, *p* < 0.05) (Figure 3B). No significant differences were noted between these groups regarding gender ratio, diagnostic delay, or the c.2238G>C allele frequency (data not shown).

**TABLE 1:** Clinical features of LOPD patients with CK < 2 UNL or CK ≥ 2 UNL.

Variables	Total (*n* = 34)	CK < 2UNL (*n* = 9)	CK ≥ 2UNL (*n* = 25)	*p* value
Gender, *n* (%)				1
Female	17 (50)	5 (56)	12 (48)	
Male	17 (50)	4 (44)	13 (52)	
Diagnostic delay, *n* (%)				1
No	13 (38)	3 (33)	10 (40)	
Yes	21 (62)	6 (67)	15 (60)	
Age at onset (y), mean ± SD	21.58 ± 9.52	31 ± 10.09	18.19 ± 6.73	0.005*
Age at diagnosis (y), mean ± SD	27.68 ± 10.03	39.67 ± 8.72	23.32 ± 6.36	<0.001*
Delay of diagnosis (y), median (Q2, Q3)	5 (1.25, 8)	7 (1, 9)	3 (2, 7)	0.433
Onset with LGMW, *n* (%)				0.034*
No	11 (32.4)	6 (50)	5 (16)	
Yes	23 (67.6)	6 (50)	17 (84)	
Onset with dyspnea, *n* (%)				0.031*
No	29 (82)	5 (50)	24 (100)	
Yes	5 (18)	5 (50)	0 (0)	
Onset with AMW, *n* (%)				1
No	30 (88)	8 (89)	22 (88)	
Yes	4 (12)	1 (11)	3 (12)	
Respiratory failure, *n* (%)				1
No	8 (24)	2 (22)	6 (24)	
Yes	26 (76)	7 (78)	19 (76)	
FVC, mean ± SD	45.32 ± 18.19	45.57 ± 14.46	45.22 ± 19.83	0.962
RV in pathology, *n* (%)				0.306
No	6 (18)	3 (33)	3 (12)	
Yes	28 (82)	6 (67)	22 (88)	
Affect siblings, *n* (%)				0.348
No	27 (79)	6 (67)	21 (84)	
Yes	7 (21)	3 (33)	4 (16)	
Spinal curvature disorders, *n* (%)				0.692
No	24 (71)	7 (78)	17 (68)	
Yes	10 (29)	2 (22)	8 (32)	
Sit‐up disability, *n* (%)				1
No	2 (7)	0 (0)	2 (11)	
Yes	25 (93)	8 (100)	17 (89)	

Abbreviations: AMW, axial muscle weakness; BMI, body mass index; CK: creatine kinase; FVC, forced vital capacity; LGMW, limb‐girdle muscle weakness; SD, standard deviation; y, year.

* *p* value<0.05.

## 4 DISCUSSION

In this study, we retrospectively explored the diagnostic journey of 34 LOPD patients. More than half (22/34) of the patients carried c.2238G>C, which is in consistency with the well accepted knowledge that c.2238G>C is the most common variant in Chinese LOPD patients. The mutational spectrum has been broadened for years. Novel mutations with milder phenotypes continued to be identified in Chinese LOPD. This indicates that China is still an underrepresented region, in which the full genetic landscape of LOPD remains uncharted. Nevertheless, the finding that hyperCKemia was rarely solely presented as an initial symptom illustrated a different early portrait of Chinese LOPD.^17^, ^18^, ^19^, ^20^, ^21^

The prompt diagnosis in patients presenting LGMW symptoms is challenging due to its non‐specificity in a variety of neuromuscular disorders and other systemic conditions. The red flag of Pompe disease would not be raised until histological studies revealed vacuolar myopathy with excessive glycogen, resulting in diagnostic delay in general approach of differentiation.^6^, ^9^, ^22^, ^23^, ^24^ Patients initially manifesting dyspnea also went through prolonged diagnostic journeys due to extensive and repeated cardiac and respiratory evaluations typically conducted by internal medicine specialists.^25^ Axial muscle weakness is easily overlooked, as it does not impact daily activities in early stage, but can be observed as a characteristic feature in over 90% of patients. Upon diagnosis, almost all the patients could recall an inability to perform sit‐ups back to early school years. Therefore, history‐taking and examination including the axial muscle domain in the initial flowchart is crucial for patients with neuromuscular symptoms. Effective implementation of high‐risk screening protocols and increased awareness among primary care givers and physicians of relevant specialties are critical to curtailing diagnostic delays.^9^

Contrary to international experiences where later onset correlates with longer diagnostic delays, in our cohort childhood‐onset LOPD cases experienced more extended diagnostic delays than adult‐onset cases.^9^ This paradox may be attributed to different hotspot mutations in the study groups. Unlike in south China where most of the childhood‐onset LOPD are carriers of the c.1935C>A variant,^15^ in our study carriers of c.2238G>C variant with the manifestation in adolescence presented with LGMW with/without hyperCKemia instead of early respiratory failure. On the other hand, compared to populations that carriers of the leaky splicing mutation IVS1 comprising the majority of adult LOPD patients, adult‐onset patients with c.2238G>C tended to develop respiratory insufficiency earlier.^17^, ^26^ Whether there are other phenotypic modifiers at play besides the hotspots remains to be elucidated.

There are several limitations to our study. It only included a small sample size and focused on adult patients with a predominance of the c.2238G>C allele frequency. It may not fully represent the diverse genetic and clinical presentations of LOPD in China. Being a cross‐sectional study, it lacks long‐term follow‐up data, which is necessary for understanding disease progression and long‐term patient outcomes. Furthermore, our study did not account for environmental factors and lifestyle variables that may influence the severity and progression of LOPD.

## 5 CONCLUSION

In conclusion, our study provided detailed information of the patient journey and discussed the risk factors of diagnostic delay. It underlies the critical need for increasing awareness of LOPD among primary care givers and physicians of relevant specialties. Recognizing early symptoms and signs such as axial muscle weakness is essential, while hyperCKemia is not always present in adult patients. The difference between childhood‐ and adult‐onset Chinese LOPD suggests to apply different screening strategy in the future. High‐risk screening using DBS for GAA enzymatic activity is crucial to facilitate timely diagnosis.

## AUTHOR CONTRIBUTIONS

Dongyue Yue and Kexin Jiao led the conceptualization and methodology planning for the study and drafted the original manuscript. They also serve as the guarantors for this article. Xingyu Xia, Jialong Zhang, and Bochen Zhu, were pivotal in data collection and analysis and contributed to the writing, review, and editing process. LingChun Liu, Kunzhao Du, Mingshi Gao, Ningning Wang, Jianying Xi, Sushan Luo, Jiahong Lu, and Nachuan Cheng were actively involved in data collection and analysis, contributing significantly to the overall study results. Finally, Wenhua Zhu and Chongbo Zhao provided supervision throughout the project, administrated the project tasks, and were involved in the manuscript's writing, review, and editing process.

## FUNDING INFORMATION

Wenhua Zhu, Jianying Xi, Chongbo Zhao, and Dongyue Yue were supported by the National Natural Science Foundation of China (82171398and 82271437); Science and Technology Commission of Shanghai Municipality (20S31904200 and 19ZR1445300).

## CONFLICT OF INTEREST STATEMENT

The authors declare that they have no conflict of interest.

## ETHICS STATEMENT

The studies involving human participants were reviewed and approved by Medical Ethics Committee of Huashan Hospital, Shanghai Medical College, Fudan University (approval no. KY2021‐537).

## INFORMED CONSENT

All procedures followed were in accordance with the ethical standards of the responsible committee on human experimentation (institutional and national) and with the Helsinki Declaration of 1975, as revised in 2000. Informed consent was obtained from all patients for being included in the study.

## Supporting information


**TABLE S1:** Clinical features and GAA mutations of 34 patients.

**TABLE S2.** Functional prediction of novel missense mutations.

## Data Availability

The data that support the findings of this study are available from the corresponding author on reasonable request.
