# Supplementary material for: Diagnostic delay in late‐onset Pompe disease among Chinese patients: A retrospective study
Source: JIMD Rep. 2023 Dec 20;65(1):39–46. doi: 10.1002/jmd2.12404 (PMC10764198; doi:10.1002/jmd2.12404)
Supplement: Supplementary file 1 — TABLE S1: Clinical features and GAA mutations of 34 patients. [file JMD2-65-39-s002.docx]

| Supplementary table1: Clinical features and GAA mutations of 34 patients | | | | | | | | | | | | | | | | | | | | | | | | | |
| --- | --- | --- | --- | --- | --- | --- | --- | --- | --- | --- | --- | --- | --- | --- | --- | --- | --- | --- | --- | --- | --- | --- | --- | --- | --- |
|  | Gender^a^ | AAO/AAD^b^ (y) | DD^c^(y) | IS^d^ | RD^e^ | **NF^f^** | **SCD^g^** | RF^h^ | Misdiagnosis | CK^i^ (Normal limit U/L) | FVC^j^(%) | EMG^k^ | MRI^l^ | RV^m^ | Biopsy Site^n^ | PC^o^ | AS^p^ | Sit-up disability | DBS^q^ | Mutation 1^j^ | | | Mutation 2^k^ | | |
|  |  |  |  |  |  |  |  |  |  |  |  |  |  |  |  |  |  |  | (pmol·punch^-1^hl^-1^)^i^ | NM_000152.5 | [NP_000143.2](https://www.ncbi.nlm.nih.gov/protein/119393891) | Loc | NM_000152.5 | [NP_000143.2](https://www.ncbi.nlm.nih.gov/protein/119393891) | Loc |
|  |  |  |  |  |  |  |  |  |  |  |  |  |  |  |  |  |  |  |  |  |  |  |  |  |  |
| 1 | F | 16/17 | 1 | LGMW | Orthopedics | 2 | － | ＋ | muscular dystrophy | 1084(134) | 30.9 | m | ND | ＋ | ND | － | － | ＋ | NA | c.1800_1823del | p.S601_R608del | exon2 | c.1634C>T | p.P545L | exon11 |
| 2 | M | 20/22 | 2 | LGMW | Emergency department | 3 | － | ＋ | muscular dystrophy, pneumonia, respiratory failure | 353(174) | NA | m | ND | ＋ | ND | － | － | NA | NA | c.1222A>G | p.P545L | exon8 | c.2214G>A | p.W738X | exon16 |
| 3 | F | 16/17 | 1 | LGMW | Rheumatology | 2- | － | ＋ | viral hepatitis | 1802(174) | 41.7 | m | ND | ＋ | QF | － | － | NA | NA | c.1634C>T | p.P545L | exon11 | c.1822C>T | p.R608X | exon13 |
| 4 | M | 24/30 | 6 | Lumbosacral pain(AMW) | Orthopaedics | 5 | ＋ | － | spinal curvature disorders, FSHD | 811(174) | NA | m | ND | ＋ | BB | － | ＋ | NA | 8 | c.796C>T | p.P266S | exon4 | c.2238G>C | p.W746C | exon16 |
| 5 | F | 6/28 | 22 | LGMW | Neurology | 2 | － | ＋ | restrictive cardiomyopathy | 600(174) | 37.1 | NA | ND | ＋ | QF | － | － | ＋ | NA | c.1634C>T | p.P545L | exon11 | c.2431delC | p.L811Wfs*37 | exon17 |
| 6 | M | 15/28 | 13 | LGMW | Gastroenterology | 2 | － | ＋ | hepatitis, myocarditis, respiratory failure | 1055(174) | NA | m | ND | ＋ | BB | － | － | ＋ | NA | c.1396delG | p.V466Ffs*11 | exon9 | c.2238G>C | p.W746C | exon16 |
| 7 | M | 16/21 | 5 | LGMW | Emergency Department | 2 | － | ＋ | myositis, progressive muscular dystrophy, respiratory failure | 1331(174) | NA | m | ND | ＋ | BB | － | － | NA | NA | c.2238G>C | p.W746C | exon16 | c.2725G>A | p.V909M | exon19 |
| 8 | F | 39/48 | 9 | LGMW | Rheumatology | 4 | － | ＋ | myositis | 164(174) | 40.1 | m | FI | ＋ | BB | － | － | ＋ | NA | c.1447G>A | p.G483R | exon10 | c.2238G>C | p.W746C | exon16 |
| 9 | M | 20/25 | 5 | LGMW | Orthopedics | 0 | － | ＋ | limb-girdle muscle syndrome | 566(174) | 15 | m | ME | ＋ | BB | － | － | NA | NA | c.2237G>A | p.W746X | exon16 | c.2238G>C | p.W746C | exon16 |
| 10 | F | 31/32 | 1 | hyperCKemia | Neurology | 5 | － | － | asymptomatic hyperCKemia | 900(174) | 90.4 | m | ND | ＋ | BB | － | － | － | 1.7 | c.-32-13T>G | IVS1 | intron 1B | c.2662G>T | p.E888X | exon19 |
| 11 | F | 20/20 | 0 | Dyspnea | Emergency Department, Neurology | NA | － | ＋ | respiratory failure | 765(174) | NA | n | ND | ＋ | BB | － | － | NA | NA | c.2105G>A | p.R702H | exon15 | c.2105G>A | p.R702H | exon15 |
| 12 | M | 20.7/21 | 0.3 | Dyspnea | Emergency Department, Gastroenterology | 0 | － | ＋ | chronic colitis, respiratory failure | 709(174) | 20 | m | ME | ＋ | DM | － | － | ＋ | 2.23 | c.1320-1322del | p.M439del | exon 8 | c.2238G>C | p.W746C | exon16 |
| 13 | F | 23/30 | 7 | LGMW | Psychiatry, emergency department | 2 | － | ＋ | depression, respiratory failure | 139(174) | 27 | m | FI | ＋ | BB | － | － | ＋ | 2.86 | c.1935C>A | p.D645E | exon14 | c.2238G>C | p.W746C | exon16 |
| 14 | M | 14/17 | 3 | LGMW | Neurology | 2 | ＋ | ＋ | MG | 1991(174) | 43 | m | ME | － | BB | － | － | ＋ | 2.92 | c.1935C>A | p.D645E | exon14 | c.2238G>C | p.W746C | exon16 |
| 15 | M | 28/35 | 7 | Spinal rigidity(AMW) | Gastroenterology, respiratory medici/ne,caedimatology | 3 | ＋ | ＋ | congenital muscular dystrophy | 742(174) | 31.8 | m | FI | ＋ | ND | － | ＋ | ＋ | 0.74 | c.-32-13T>G | IVS1 | intron 1B | c.307T>C | p. C103R | exon2 |
| 16 | M | 26/33 | 7 | LGMW | Neurology | 2 | ＋ | ＋ | ankylosing spinal syndrome | 447(174) | 32.3 | m | ME | ＋ | ND | － | － | ＋ | NA | c.1320-1322del | p.M440del | exon 8 | c.2238G>C | p.W746C | exon16 |
| 17 | M | 3/13 | 10 | LGMW | Neurology | 1 | ＋ | ＋ | / | 885(174) | 57.3 | m | ME | － | BB | － | － | ＋ | NA | c.2815_2816del | p.V939Lfs*78 | exon20 | c.2238G>C | p.W746C | exon16 |
| 18 | M | 24/26 | 2 | LGMW | Orthopaedics | 4 | － | ＋ | spinal curvature disorders | 1247(174) | 51.9 | m | ND | － | BB | － | － | ＋ | NA | c.2238G>C | p.W746C | exon16 | c.2238G>C | p.W747C | exon16 |
| 19 | F | 24/26 | 2 | LGMW | Orthopaedics | 2 | － | － | osteoarthritis, MG | 639(174) | 50 | normal | ME | ＋ | ND | － | － | ＋ | 0.36 | c.1562A>T | P.E521V | exon11 | c.2173C＞T | p.R725T | exon15 |
| 20 | M | 16/18 | 2 | LGMW | Orthopaedics | 1 | ＋ | ＋ | hysteria, muscular dystrophy | 1089(200) | 44.6 | m | ME | ＋ | ND | － | － | ＋ | 0.55 | c.2237G>A | p.W746X | exon16 | c.2238 G>C | p.W746C | exon16 |
| 21 | F | 39/47 | 8 | LGMW | Orthopaedics | 3 | ＋ | － | spinal curvature disorders, polymyelitis | 229(174) | 46.3 | m | ND | － | BB | － | ＋ | ＋ | 2.23 | C.2297A>G | p.Y766C | exon16 | c.1879_1881del | p.S627del | exon13 |
| 22 | F | 25/30 | 5 | LGMW | Neurology | 2 | － | ＋ | peripheral neuropathy | 391(174) | 45.7 | m | normal | ＋ | ND | － | － | ＋ | NA | c.1928G>C | p.G643A | exon14 | c.2237G>A | p.W746X | exon16 |
| 23 | M | 7/15 | 8 | hyperCKemia | Neurology | 2 | － | － | asymptomatic hypercKemia, muscular dystrophy | 2659(174) | 36.2 | m | ME | ＋ | ND | － | ＋ | ＋ | 0.3 | c.1935C>A | p.D645E | exon14 | c.2238G>C | p.W746C | exon16 |
| 24 | M | 10/35 | 25 | LGMW | respiratory medicine, emergency department | 4- | － | ＋ | pulmonary tuberculosis, intestinal tuberculosis,pneumonia. respiratory failure | 78(174) | NA | m | ND | ＋ | BB | － | － | ＋ | NA | c.925G>A | p.G309R | exon5 | c.2238G>C | p.W746C | exon16 |
| 25 | M | 17/27 | 10 | AMW | Neurology | 3 | ＋ | ＋ | muscular dystrophy | 816(174) | 32.1 | m | ME | ＋ | ND | ＋ | － | ＋ | NA | c.1316T>A | p.M439K | exon8 | c.1316T>A | p.M439K | exon8 |
| 26 | M | 30.5/31 | 0.5 | Dyspnea | emergency department | 3 | － | ＋ | pneumonia, respiratory failure | 148(174) | NA | m | normal | ＋ | ND | － | ＋ | NA | NA | c.953T>A | p.M318K | exon5 | c.953T>A | p.M318K | exon5 |
| 27 | F | 23/26 | 3 | LGMW | Orthopedics | 2 | － | － | myositis | 2492(174) | 79.3 | m | ME | ＋ | ND | － | － | － | 0.51 | c.546G>T | p.[=,0, Ile183Valfs*67] | exon2 | c.1669A>T | p.I557p | exon2 |
| 28 | F | 27.5/28 | 0.5 | Dyspnea | Cardiology, emergency department | 2 | － | ＋ | pulmonary arterial hypertension , respiratory failure | 182(174) | 38.4 | m | ME | ＋ | ND | － | － | ＋ | 0.22 | c.2238G>C | p.W746C | exon16 | C.2800-1G>T | ? | intron19 |
| 29 | F | 36/43 | 7 | LGMW | Gastroenterology, Psychiatry, emergency department, respiratory medicine | 2+ | － | ＋ | gastritis, depression, respiratory failure | 151(180) | 58.5 | normal | FI | ND | ND | － | － | ＋ | 0.19 | c.2238G>C | p.W746X | exon16 | c.2238G>C | p.W746C | exon16 |
| 30 | F | 43/44 | 1 | Dyspnea | Orthopedics，respiratory medicine | 0 | ＋ | ＋ | spinal curvature disorders, respiratory failure | 258(174) | 39.1 | m | normal | － | BB | ＋ | － | ＋ | 0.19 | c.2238G>C | p.W746X | exon16 | c.2238G>C | p.W747C | exon16 |
| 31 | M | 31/51 | 20 | Waddling gait | Neurology | 2 | － | － | myositis | 281(174) | 70.4 | m | ME | ＋ | TA | ＋ | ＋ | ＋ | 0.18 | c.568C>G | p.R190G | exon3 | c.1082C>T | p.P361L | exon7 |
| 32 | F | 11/15 | 4 | LGMW | emergency department | 2 | － | ＋ | pneumonia | 1209(174) |  |  | normal | ＋ | ND | － | ＋ | ＋ | 0.55 | c.1411_1414del;  [c.752C>T;c.761  C>T] | p.E471fs;  p.[S251L; S254L] | exon9; exon4 | c.1444C>T | p.P482S | exon10 |
| 33 | F | 15/16 | 1 | LGMW | Neurology | 2 | － | － | myositis | 1256(174) | 75.2 | m | ME | ＋ | BB | － | － | ＋ | 0.72 | c.2238G>C | p.W746C | exon16 | c.1082C>T | p.P361L | exon7 |
| 34 | F | 15/26 | 11 | LGMW | respiratory medicine, Chinese traditional medicine, Orthopaedics,Gastroenterology | 1 | ＋ | ＋ | / | 508(174) | 14,6 | m | MA | ＋ | BB | － | － | ＋ | 0.39 | c.1798C>T | p.R600C | exon13 | c.521A>G | p.E174G | exon2 |
| Abbreviations: | | | | | | | | | |  |  |  |  |  |  |  |  |  |  |  |  |  |  |  |  |
| a. F, Female; M: Male | | | | | | | | | |  |  |  |  |  |  |  |  |  |  |  |  |  |  |  |  |
| b. AAO/AAD: Age at onset/Age at diagnosis; | | | | | | | | | |  |  |  |  |  |  |  |  |  |  |  |  |  |  |  |  |
| c. DD: Differential Diagnosis; | | | | | | | | | |  |  |  |  |  |  |  |  |  |  |  |  |  |  |  |  |
| d. IS: Initial Symptom; LGMW: Limb-girdle muscle weakness | | | | | | | | | |  |  |  |  |  |  |  |  |  |  |  |  |  |  |  |  |
| e. RD: Referral Department; | | | | | | | | | |  |  |  |  |  |  |  |  |  |  |  |  |  |  |  |  |
| f. NF: Neurological Findings; | | | | | | | | | |  |  |  |  |  |  |  |  |  |  |  |  |  |  |  |  |
| g. SCD: Spinal Curvature Disorders; | | | | | | | | | |  |  |  |  |  |  |  |  |  |  |  |  |  |  |  |  |
| h. RF: Respiratory Failure; | | | | | | | | | |  |  |  |  |  |  |  |  |  |  |  |  |  |  |  |  |
| i. CK: Creatine Kinase; | | | | | | | | | |  |  |  |  |  |  |  |  |  |  |  |  |  |  |  |  |
| j. FVC: Forced Vital Capacity; | | | | | | | | | |  |  |  |  |  |  |  |  |  |  |  |  |  |  |  |  |
| k. EMG: Electromyography | | | | | | | | | |  |  |  |  |  |  |  |  |  |  |  |  |  |  |  |  |
| l. MRI: Magnetic Resonance Imaging; | | | | | | | | | |  |  |  |  |  |  |  |  |  |  |  |  |  |  |  |  |
| m. RV: Respiratory Volume;NA: Not Available or Not Applicable; ND: Not Done or Not Determined | | | | | | | | | |  |  |  |  |  |  |  |  |  |  |  |  |  |  |  |  |
|  | | | | | | | | | |  |  |  |  |  |  |  |  |  |  |  |  |  |  |  |  |
|  | | | | | | | | | |  |  |  |  |  |  |  |  |  |  |  |  |  |  |  |  |
